# The Relationship of Nest‐Site Selection Parameters, Timing of Breeding, Brood Size, and Nestling Body Condition With Brood Sex Ratio in the Black‐Crowned Night Heron *Nycticorax nycticorax*

**DOI:** 10.1002/ece3.73737

**Published:** 2026-06-03

**Authors:** Seyedeh Farahnaz Vesali, Hossein Varasteh Moradi, Seyed Mehdi Amininasab, Zarbakht Ansari Pirsaraei

**Affiliations:** ^1^ Environmental Engineering and Sciences, Faculty of Fisheries and Environmental Sciences Gorgan University of Agricultural Sciences and Natural Resources Gorgan Iran; ^2^ Department of the Environmental Sciences, Faculty of Fisheries and Environmental Sciences Gorgan University of Agricultural Sciences and Natural Resources Gorgan Iran; ^3^ Department of Natural Resources, Faculty of Natural Resources Sari Agricultural Sciences and Natural Resources University Sari Iran; ^4^ Department of Animal Science, Faculty of Animal Science and Fisheries Sari Agricultural Sciences and Natural Resources University Sari Iran

**Keywords:** Ardeidae, fitness, reproductive success, sex allocation

## Abstract

Brood sex ratio is a fundamental aspect of evolutionary biology, with profound implications for life‐history traits, parental investment, sex allocation, offspring rearing, and ultimately, reproductive success, population dynamics, and viability. Previous research on birds brood sex ratio bias has demonstrated associations with various parameters, including environmental and reproductive conditions, the social environment, and parental quality. Identifying and understanding the parameters that influence brood sex ratio can significantly advance our knowledge of sex allocation in ecological and evolutionary contexts. The Black‐crowned Night Heron *Nycticorax nycticorax* , a colonial species in the family Ardeidae, is sexually monomorphic, making it an ideal biological model for sex allocation studies. The present study, conducted in 2023, assessed the relationships between the brood sex ratio and several variables: nest‐site selection parameters (nests = 18), timing of breeding, brood size, and nestling body condition. Research was carried out at the Zaghmarz Forest and Rangeland Research Station, an anthropogenic ecosystem dedicated to silvicultural research in eastern Mazandaran Province, northern Iran. Blood samples were collected from nestlings (*n* = 54) to determine the sex of each individual. The findings revealed that none of the investigated parameters—nest‐site selection, timing of breeding, brood size, or nestling body condition—had a significant relationship with the brood sex ratio. These results suggest that the brood sex ratio in Black‐crowned Night Herons may be primarily controlled by genetic mechanisms or other unmeasured physiological and environmental parameters. This underscores the need for further research into the mechanisms regulating brood sex ratio in this species.

## Graphical abstract

This study investigated the relationship of nest‐site selection parameters, timing of breeding, brood size, and nestling body condition with the brood sex ratio of Black‐crowned Night Herons ( *Nycticorax nycticorax* ) in northern Iran during 2023. Nestling sex was determined via PCR amplification of the CHD gene from blood samples. Results showed no significant relationship between brood sex ratio with these parameters, indicating that sex ratio may be governed by genetic or other unexamined mechanisms, highlighting the need for further research on sex allocation in avian species.

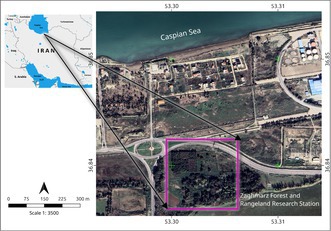

## 1 Introduction

In birds, the brood sex ratio is defined as the proportion of males relative to the total number of nestlings (Louder et al. 2020). This metric has garnered significant attention in behavioral ecology as a means of understanding strategies related to parental investment and sex allocation (Bartlow et al. 2021). Understanding these ratios has important implications for population dynamics, stability, and conservation (Ewen et al. 2001; Alonso‐Alvarez 2006). Biased sex ratios present a common challenge in both wild bird populations and captive breeding programs, particularly for threatened species (Lenz et al. 2007; Brooke et al. 2010). According to Fisher's principle (1930), if the costs and benefits of producing male and female offspring are equal, brood sex ratios at the population level are expected to be balanced (i.e., 1:1) (Ewen et al. 2001). However, this equilibrium is not always achieved; consequently, parents may invest differentially—either by altering brood sex ratios or by varying energy allocation between male and female offspring—to maximize their reproductive success based on prevailing environmental conditions (Trivers and Willard 1973; Charnov 1982; Ligon and Hill 2010).

The Trivers–Willard hypothesis predicts that when one sex (typically males) exhibits greater variation in reproductive value, female parents in good condition should invest more in male offspring, while those in poor condition should direct greater investment toward female offspring to maximize their own reproductive success (Veller et al. 2016). Because the reproductive value of offspring can vary due to a range of environmental and biological parameters, females should adjust the brood sex ratio accordingly (Sheldon 1998; Nager et al. 1999; Calsbeek and Sinervo 2004).

One key parameter influencing the brood sex ratio is the structure and characteristics of the nest site (Cresswell 2011). By affecting access to food resources and other environmental conditions, nest sites play a critical role in rearing success and species survival (Martin 1998). It is predicted that larger nests—often indicative of higher‐quality individuals—will exhibit biased brood sex ratios (Fargallo et al. 2004). This is likely because, in some bird species, male nestlings are slightly larger than females and thus require more nest space (Spelt and Pichegru 2017). Among breeding birds, especially colonial species, higher nest density and shorter inter‐nest distances may enhance group defense and anti‐predator behaviors (Anderson and Hodum 1993), which are also predicted to influence brood sex ratios (see Dorr et al. 2014; Minias et al. 2014). Furthermore, greater nest height above the ground protects eggs and chicks from terrestrial predators, thereby improving overall breeding success (Bachir et al. 2008; Abdullah et al. 2017). Consequently, biased brood sex ratios are expected in nests located at higher elevations (see Gómez‐López et al. 2023). Additionally, larger trees may influence resource access or microhabitat features, potentially leading to biased ratios in nests built upon them (see Suorsa et al. 2004; Székely et al. 2014). Birds further enhance their reproductive success by selecting optimal nest positions for their structural stability and protective advantages; experienced, high‐quality pairs typically occupy the best sites (Bachir et al. 2008; Minias et al. 2014), which is believed to influence brood sex ratio bias.

Phenology or timing of breeding is another parameter that influences the brood sex ratio (Radford and Blakey 2000; Bartlow et al. 2021). Early‐season breeding is considered an indicator of high adult quality and reflects a superior ability to provide effective parental care. However, early spring also brings weather anomalies and the risk of chilling (Tobolka et al. 2015). As the weather becomes more stable later in the season, the nestlings of these early breeders hatch earlier, providing them with better access to food resources and improved growth opportunities (Schreven et al. 2022). In contrast, nestlings that hatch late in the season often exhibit accelerated growth rates and attain flight capability at younger ages, which can ultimately reduce their survival and reproductive success (Kasprzykowski et al. 2014). Some studies have indicated that early in the breeding season, brood sex ratios in the Northern House Wren *Troglodytes aedon* are significantly male‐biased, an adjustment associated with optimal environmental conditions and high food abundance that enhances male survival (Bowers et al. 2014).

Brood size, which correlates with clutch size, also influences the brood sex ratio (Bartlow et al. 2021). A relationship often exists between these two variables because females in superior physiological condition may produce more nestlings with biased sex ratios. For instance, research on the Northern House Wren has shown a significant positive relationship between clutch size and a higher proportion of males in a brood (Bowers et al. 2014).

Another key parameter in brood sex ratio adjustment is variation in nestling body quality or condition (Vedder et al. 2005; Saino et al. 2008). Morphometric indices—including body, wing, and tarsus length, as well as body mass—are commonly used as proxies for assessing physical condition (Labocha and Hayes 2012). Brood sex ratios are predicted to depend on these indices (Vedder et al. 2005), with biased ratios expected among nestlings in better body condition (see Rosivall et al. 2010; Bowers et al. 2015; Gyarmathy et al. 2024).

Identifying the key parameters that drive sex allocation remains challenging. Results regarding brood sex ratios are frequently contradictory, both within and between species (Michler et al. 2013). To date, most studies on sex ratio adjustment have focused on terrestrial species, particularly those with sexual size dimorphism, where one sex is larger and more costly to produce. Fewer studies have addressed sex ratio deviations in waterbirds, especially among sexually monomorphic species (Bartlow et al. 2021).

Due to its colonial breeding habits and the accessibility of large brood samples, the Black‐crowned Night Heron *Nycticorax nycticorax* is an ideal biological model for research. It is a sexually monomorphic waterbird species within the family Ardeidae. To date, however, no study has examined the parameters influencing brood sex ratio variation within this family. Therefore, this study aims to determine the relationships between nest‐site selection, timing of breeding, brood size, body condition, and the brood sex ratio of this species. We hypothesize that brood sex ratios will be male‐biased in high‐quality nests, characterized by larger diameters and depths (see Fargallo et al. 2004), higher nest density and shorter inter‐nest distances (see Dorr et al. 2014; Minias et al. 2014), greater height from the ground (see Gómez‐López et al. 2023), placement on trees with larger trunk circumferences (see Suorsa et al. 2004), and proximity to the tree trunk (see Bachir et al. 2008; Minias et al. 2014). We also predict male‐biased brood sex ratios in early‐hatching broods (see Bednarz and Hayden 1991; Bowers et al. 2014), in nests with larger brood sizes (see Bowers et al. 2014), and among nestlings in superior physical condition (as indicated by body mass and body, wing, and tarsus lengths) (see Rosivall et al. 2010; Bowers et al. 2015; Gyarmathy et al. 2024). However, previous findings on the relationships between these parameters and brood sex ratios remain contradictory; thus, the current study aims to address the existing knowledge gap regarding the parameters influencing brood sex ratios in birds.

## 2 Methods

### 2.1 Study Area

**FIGURE 1:**
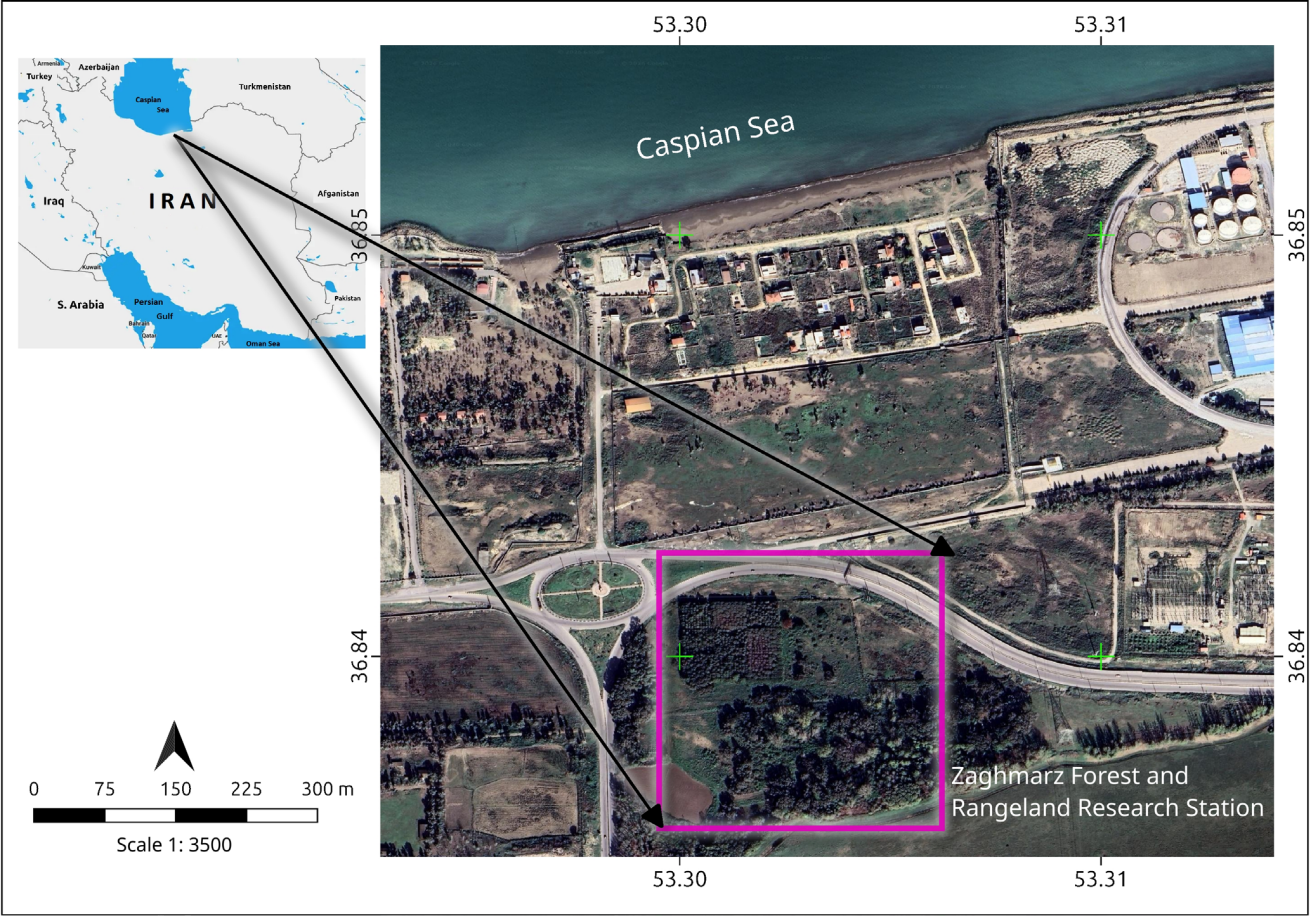
Location map of the Zaghmarz Forest and Rangeland Research Station study area in Mazandaran Province, northern Iran.

This study was conducted at the Zaghmarz Forest and Rangeland Research Station in Mazandaran Province, northern Iran (36°50′35″ N, 53°18′15″ E) (Figure 1). The study area, an anthropogenic ecosystem, is located 1 km from the southern Caspian Sea coast at approximately 20 m below sea level, situated west of the international Miankaleh and Lapoo‐Zaghmarz wetlands. Coniferous trees—specifically Turkish pine *Pinus brutia* , longleaf Indian pine *Pinus roxburghii*, and stone pine *Pinus pinea* —have been planted at a 3 × 3 m spacing since 1997 for silvicultural research (Khorankeh et al. 2014). The station also provides nesting habitat for members of the Ardeidae family, including the Little Egret *Egretta garzetta* , Black‐crowned Night Heron *Nycticorax nycticorax* , Cattle Egret *Bubulcus ibis* , and Squacco Heron *Ardeola ralloides* . During the study, there were no active human disturbances, such as visitation or land use, that might have affected the nesting birds.

### 2.2 Nest and Brood Selection and Monitoring

Active nests within the Black‐crowned Night Heron colony were identified through direct observation. Initially, a subset of 30 trees was randomly selected for continuous monitoring starting from the onset of egg‐laying (20 April 2023), with each nest assigned a unique numerical identifier. These nests were inspected every 3–5 days during the incubation period until the first hatching occurred on 24 May 2023. This inspection interval was chosen to minimize disturbance; visits were conducted only under favorable weather conditions (avoiding rain or cold) and were performed to mitigate the risks of premature fledging or predation.

Following the initial hatching, nests were monitored daily until the completion of the fledging stage (20 June 2023). To standardize the timeline, the date of the first hatching event (24 May 2023) was designated as Day 1. Nests were included in the final analysis only if 100% of the eggs hatched successfully and produced nestlings. To ensure the sample was representative of the colony, only one nest per tree was selected, resulting in a final sample size (*n* = 18). For each nest, clutch size (total eggs laid) and brood size (number of nestlings) were recorded following established protocols for heron research (Bowers et al. 2015; Bartlow et al. 2021).

### 2.3 Nest Selection Parameters and Measurements

Nest selection parameters were measured for each nest (*n* = 18) by accessing the nests using ladders and ropes, in accordance with the criteria specified above. Measurements were taken during the chick‐rearing stage through direct observation. Nest parameters and substrate tree characteristics were recorded using metal tapes. The parameters included the nest substrate species (Kannan and Pandiyan 2012), the total number of nests on the substrate tree (Kazantzidis et al. 1997; Roshnath and Sinu 2017), and the tree's circumference at breast height (CBH, cm) (Roshnath and Sinu 2017). Nest diameter (cm) and depth (cm) (Yu and Hahm 1997; Abdullah et al. 2017), the distance to the nearest neighboring nest (cm) (Kazantzidis et al. 1997), and nest height above the ground (m) (Samraoui et al. 2025) were measured as well. Finally, the horizontal position of each nest was categorized based on direct field observations into three types: placement on the main trunk, on branches near the trunk, or on branches away from the trunk (Kazantzidis et al. 1997).

### 2.4 Measurement of Nestlings Morphometric Parameters

Nestlings were transferred from the nest to the ground in cloth bags for standard morphometric measurements. Handling was kept to a minimum (< 5 min per individual) and restricted to nestlings aged 5–7 days; individuals were returned immediately to the nest to prevent chilling or parental desertion. Morphometric parameters for the nestlings (*n* = 54)—including body mass (kg), body length (cm), wing length (cm), and tarsus length (cm)—were recorded as indices of body condition (Labocha and Hayes 2012; Sandvig et al. 2022; Gajdošová et al. 2023). These measurements were obtained using a hanging digital balance, a metal ruler, and calipers, respectively. Because hatching is asynchronous, nestlings of varying sizes were present within the same nest. However, using the average of the aforementioned body condition parameters for nestlings aged 5–7 days likely accounts for this variation. Furthermore, a smaller size does not necessarily indicate that a nestling is in poorer physical condition. No incidents of accidental mortality occurred during the measurement process.

### 2.5 Blood Sampling and Sample Storage Methods

Following the recording and measurement of the nestlings' morphometric parameters described previously, nestling blood sampling was conducted via minimally invasive brachial or jugular venipuncture (26–30 gauge needles), limited to ≤ 1% body mass (~10 μL per individual). A small hole was punctured in the brachial vein of each nestling using a fine syringe needle, approximately 10 μL of blood was collected using capillary tubes, and transferred to plastic microtubes containing 96% ethanol. Once blood was drawn, the puncture site was disinfected and covered with sterile cotton to ensure hemostasis. All procedures were completed within five minutes per nestling to minimize stress; no adverse effects on nest success, fledging success, or nestling survival were observed. The blood samples were stored at 4°C until DNA extraction and sex determination (Baeta et al. 2012; Kamiński et al. 2015).

### 2.6 DNA Extraction Using the Salting‐Out Method

DNA was extracted from ethanol‐preserved blood samples using the salting‐out method (Aljanabi and Martinez 1997). To isolate the required blood, the ethanol in the microtube was transferred to a separate tube. Then, 10–15 μL of the settled blood remaining in the microtube (which still contained traces of ethanol) was pipetted into a new microtube. To evaporate the remaining ethanol, which can negatively affect DNA extraction, the microtubes containing the samples were placed in a heating block at 55°C. The following reagents were added to each sample: 30 μL proteinase K, 410 μL extraction buffer (containing Tris–HCl, NaCl, EDTA, and water), and 80 μL of 10% SDS. The resulting solution was thoroughly mixed and incubated in a water bath at 37 or 55°C for 12–24 h to ensure complete digestion. After this step, the samples were vortexed for 30 s and centrifuged at 13,000 rpm for 5 min to separate cellular debris. The transparent supernatant was transferred to a new sterile microtube containing 180 μL of NaCl solution. The contents of the microtube were mixed, vortexed for 30 s, and centrifuged at 13,000 rpm for 5 min. DNA was then precipitated by transferring the supernatant to a new microtube and adding 420 μL of cold isopropanol (−21°C). It should be noted that no vortexing is required at this stage. The DNA was pelleted by gentle mixing through five inversions of the tube followed by centrifugation at 13,000 rpm for 5 min, after which the supernatant was discarded. The DNA pellet was washed twice with 250 μL of 80% ethanol; each time, after vortexing for 30 s and centrifugation at 13,000 rpm for 5 min, the supernatant was removed. To ensure complete removal of residual ethanol, the microtubes were dried under vacuum. After complete evaporation of ethanol, 20–50 μL of sterile nuclease‐free water was added to dissolve the DNA at 37°C. The extracted DNA was stored at −20°C for long‐term preservation (Aljanabi and Martinez 1997).

### 2.7 Molecular Sexing of Nestlings via CHD‐PCR

In this study, following DNA extraction from the samples, the CHD genes linked to DNA helicase on the Z and W sex chromosomes were amplified using the polymerase chain reaction (PCR) technique with P2 and P8 primers, enabling visualization of the results via agarose gel electrophoresis (Griffiths et al. 1998). The PCR process involved denaturation of double‐stranded DNA by heat, annealing of primers to complementary regions on the single‐stranded DNA at a specific temperature, and extension of DNA fragments by the responsible polymerase enzyme; this cycle was repeated multiple times to generate sufficient product for detection on agarose gel. The PCR thermal cycling conditions consisted of an initial denaturation at 94°C for 4 min, followed by 35 cycles of 94°C for 30 s, 52°C for 45 s, and 72°C for 45 s, a final extension at 72°C for 5 min, and a hold at 12°C for 1 s. PCR products were stained and visualized under UV light following electrophoresis on 3% agarose gels. Samples with a single band were scored as male and those with two bands as female (Bonderud et al. 2017; Çakmak et al. 2017). Ambiguous cases were re‐amplified to confirm sex. Molecular sexing accuracy was verified by repeating CHD‐PCR (P2/P8) on ~10% random subset (*n* = 10), all confirming original results (Fridolfsson and Ellegren 1999).

### 2.8 Statistical Methods

**FIGURE 2:**
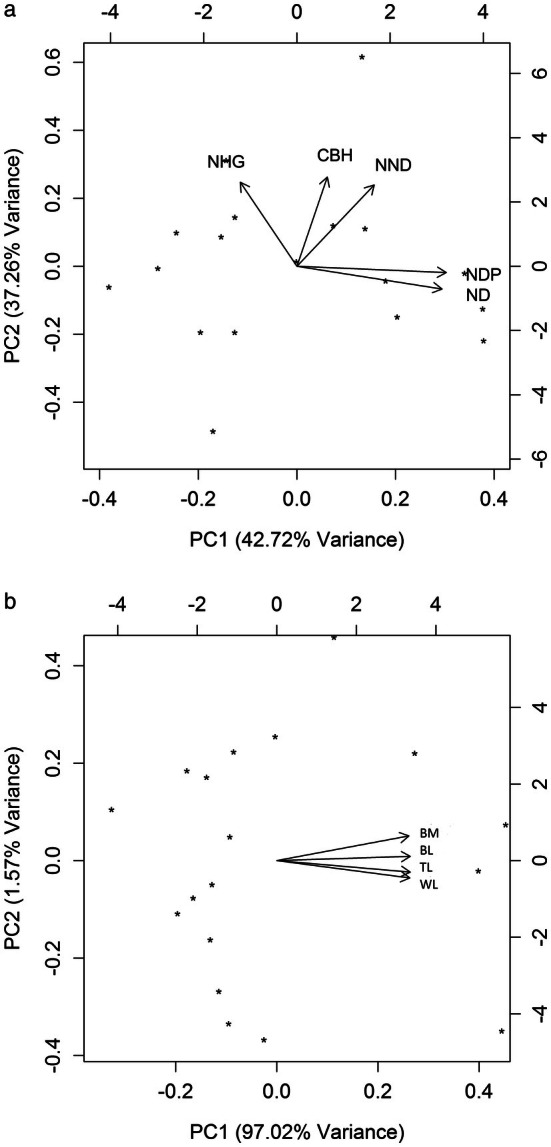
Principal component analysis (PCA) biplots for the Black‐crowned Night Heron: (a) nest parameters, including nest diameter (ND), nest depth (NDP), distance to the nearest nest (NND), nest height above the ground (NHG), and trunk circumference at breast height (CBH) of the substrate tree. PC1 and PC2 explained 42.72% and 37.26% of the total variance, respectively. (b) Nestling body condition parameters, including body mass (BM), wing length (WL), body length (BL), and tarsus length (TL). PC1 and PC2 explained 97.02% and 1.57% of the total variance, respectively.

Following data preparation, variable normality was evaluated using Q‐Q plots (via the ‘qqnorm’ function). Principal component analysis (PCA; ‘prcomp’ function) was used to examine correlations between measured nest‐site parameters and nestling body condition, identifying key variables and reducing dimensionality for modeling. Pearson correlation tests (‘cor.test’ function) were also employed to assess the relationships between these parameters. Results from the PCA and Pearson correlation tests for nest parameters (Figure 2a, Table S1) showed that nest depth and diameter were significantly and positively correlated; consequently, nest diameter was selected as the representative parameter. Similarly, distance to the nearest nest and trunk circumference at breast height (CBH) were positively correlated, with CBH chosen as the representative variable. Nest height above the ground was included in the model as it showed no correlation with other parameters. Based on the PCA and Pearson correlation coefficients for nestling body condition (Figure 2b, Table S2), all body condition parameters were highly correlated. Therefore, body mass was selected as the representative metric for nestling body condition (from among body mass, wing length, body length, and tarsus length) for model inclusion.

Generalized linear models (GLMs) with a negative binomial distribution (using the ‘glm’ function) were used to examine how predictor variables influenced the brood sex ratio of Black‐crowned Night Herons (the full model). Predictors included selected nest‐site parameters (nest diameter, CBH, and nest height above the ground, along with the number of nests per tree and nest position), timing of breeding, brood size, and nestling body condition (body mass). To identify the best‐supported model, automated stepwise model selection was performed using the ‘step’ function (Table 1). This selection was guided by the Akaike Information Criterion (AIC), starting from the full model containing all predictive variables. The final best‐fit model was selected based on the minimum AIC value (Delta AIC = 0; ‘AIC’ function). To assess explanatory power, McFadden's Pseudo‐*R* ^2^ was calculated (using the ‘update’ and ‘logLik’ functions), where values between 0.2 and 0.4 indicate an excellent model fit.

**TABLE 1:** Results of the model selection—including the full, intermediate, and best generalized linear models (negative binomial distribution)—examining the relationship of nest, breeding, and nestling body condition parameters with the brood sex ratio of Black‐crowned Night Herons are presented.

Model	Formula (predictors)	*K*	AIC	Delta AIC	McFadden's Pseudo‐*R* ^2^
Full Model	Nest diameter + Nest height above the ground + Number of nests per nest tree + Timing of breeding + Brood size + Nestlings' body mass + Circumference of the trunk at breast height + Nest position	9	38.15	8.51	0.26
Intermediate	Nest diameter + Nest height above the ground + Number of nests per nest tree + Timing of breeding + Brood size + Nestlings' body mass + Nest position	8	36.13	6.49	0.26
	Nest diameter + Nest height above the ground + Number of nests per nest tree + Brood size + Nestlings' body mass + Nest position	7	34.13	4.49	0.26
	Nest diameter + Nest height above the ground + Number of nests per nest tree + Nestlings' body mass + Nest position	6	32.27	2.63	0.25
	Nest diameter + Nest height above the ground + Number of nests per nest tree + Nest position	5	30.49	0.85	0.24
	Nest diameter + Nest position + Nest height above the ground	4	28.46	1.18	0.24
Best Model	Nest diameter + Nest position	3	29.64	0	0.11

Abbreviations: AIC, Akaike information criterion; Delta AIC,difference in AIC relative to the best model; K, number of parameters; McFadden's Pseudo‐*R* ^2^, goodness‐of‐fit measure for the selected models.

McFadden's Pseudo‐*R* ^2^ values indicated an excellent fit for both the full and intermediate models (Table 1). The most parsimonious model (Delta AIC = 0) suggested that only nest diameter and nest position explained variation in the brood sex ratio. However, the *p*‐values for nest diameter (estimate = −0.096, SE = 0.084, *z* = −1.134, *p* = 0.257), ‘far trunk’ position (estimate = 2.858, SE = 1.830, z = 1.562, *p* = 0.118), and ‘main trunk’ position (estimate = 0.227, SE = 1.498, z = 0.151, *p* = 0.880) in this model revealed no significant relationships. Furthermore, the McFadden's Pseudo‐*R* ^2^ for this final model was relatively low (0.11), suggesting limited explanatory power compared to the full model (Table 1). Consequently, the results of the full model—which includes eight predictors related to the nest‐site, breeding, and nestling body condition—are presented as the final results for interpretation. All statistical analyzes were conducted in R software using the stats package (version 4.4.2; R Core Team 2024).

## 3 Results

Means and standard deviations for the measured nest‐site, breeding, nestling body condition, and brood sex ratio parameters are presented in Table 2. In total, 38 male and 16 female nestlings were recorded across the 54 sampled nests. The full generalized linear model (GLM) indicated no significant relationships between the brood sex ratio and any of the predictor variables, including nest characteristics (nest diameter, nest height above the ground, circumference of the trunk at breast height, number of nests per nest tree, nest position), timing of breeding, brood size, and nestlings' body mass (Table 3).

**TABLE 2:** Descriptive statistics of nest, breeding, and nestling body condition parameters of Black‐crowned Night Heron.

Factors	Parameters	Minimum–Maximum	Mean ± SD
Nest (*n* = 18)	Nest diameter (cm)	25–52	34.722 ± 8.574
	Nest depth (cm)	3–11	7.167 ± 2.307
	Nearest neighbor nest distance (cm)	30–140	75.056 ± 27.074
	Nest height above the ground (m)	5.55–8.4	6.811 + 0.827
	Number of nests per tree	4–28	18.611 ± 7.823
	Circumference of the trunk at breast height (cm)	45–119	75.861 ± 15.635
Breeding (*n* = 18)	Timing of breeding (1 = 24 May 2023)	1–11	6.556 ± 3.485
	Clutch size	2–4	3.222 ± 0.647
	Brood size	2–4	3.000 ± 0.767
Nestling body condition (*n* = 54)	Body mass (g)	20–470	179 ± 149
	Body length (cm)	10.2–34.8	21.733 ± 6.679
	Wing length (cm)	3.7–26.6	11.883 ± 7.076
	Tarsus length (cm)	1.6–7.8	4.178 ± 1.711
Brood sex ratio (*n* = 54)	Number of nestling males	0–4	2.111 ± 1.323
	Number of nestling females	0–3	0.889 ± 1.023
	Proportion of males (number of males/brood size)	0–1	0.676 ± 0.371

**TABLE 3:** Full generalized linear models with negative binomial distribution examining the relationships of nest, breeding, and nestling body condition parameters with the brood sex ratio of Black‐crowned Night Heron.

	Estimate	Standard error	*Z*	*p*
Intercept	6.853	19.117	0.358	0.720
Nest diameter	−0.152	0.182	−0.832	0.405
Nest height above the ground	−0.655	1.514	−0.432	0.665
Circumference of the trunk at breast height	0.005	0.056	0.081	0.935
Number of nests per nest tree	0.035	0.108	0.327	0.744
Nest position, Far trunk	2.850	1.905	1.496	0.135
Nest position, Main trunk	0.096	2.620	0.037	0.971
Timing of breeding	0.057	0.610	0.093	0.926
Brood size	0.269	1.237	0.218	0.828
Nestlings' body mass	3.349	16.157	0.207	0.836

## 4 Discussion

### 4.1 Nest Site Selection Parameters and Brood Sex Ratio

Contrary to our expectations, the lack of a significant relationship between nest spatial variables—including nest diameter, distance to the nearest nest, nest height above the ground, trunk circumference at breast height (CBH), the number of nests on the host tree, and nest position—and Black‐crowned Night Heron brood sex ratios suggests that these parameters may not serve as reliable direct cues or constraints for parental sex allocation (Nager et al. 1999; Rosivall et al. 2010; Szász et al. 2017, 2023). Furthermore, these variables do not appear to be consistent determinants across bird species (see Fargallo et al. 2004 for similar findings; see Dubois et al. 2006; Minias et al. 2014; Rubalcaba and Polo 2022; Wells et al. 2024 for contrasting results). Nests were found exclusively on stone pines, with none observed on longleaf Indian pines, which precluded a comparison of brood sex ratios between tree species. This nesting pattern may stem from the delayed arrival of Black‐crowned Night Herons at the breeding site relative to other heron species, or from a strong preference for specific tree characteristics. Overall, these findings suggest that variation in brood sex ratios is likely driven by other parameters, such as maternal physiological state (Nager et al. 1999; Whittingham et al. 2002; Song et al. 2016), parental investment strategies, food availability, or other ecological constraints (Howe 1976; Székely, Cuthill, et al. 2004; Székely, Freckleton, and Reynolds 2004).

### 4.2 Timing of Breeding and Brood Sex Ratio

In the present study, no relationship was observed between the timing of breeding and the brood sex ratio in the Black‐crowned Night Heron (for similar findings, see Stauss et al. 2005; Ding et al. 2017; Que et al. 2019). In contrast, other studies have suggested that greater investment in costlier male (Velando et al. 2002; Székely, Cuthill, et al. 2004; Székely, Freckleton, and Reynolds 2004; Kamiński et al. 2019) or female nestlings (Louder et al. 2020; Schreven et al. 2022; Xirouchakis et al. 2023) is expected at the beginning of the breeding season, likely due to superior food availability. The absence of such a relationship in our results suggests that maternal condition and ecological or environmental constraints may be more influential drivers of brood sex ratios in certain bird species (Sandercock et al. 2005; Que et al. 2019).

### 4.3 Brood Size and Brood Sex Ratio

No relationship was observed between brood size and the brood sex ratio in the Black‐crowned Night Heron (for similar findings, see Hartley et al. 1999; Ramsay et al. 2003; Stauss et al. 2005). In contrast, other studies have suggested that a significant bias toward the smaller sex (e.g., males in raptors or females in certain passerines) is expected in the largest broods of sexually dimorphic species (Dijkstra et al. 1998; Dyrcz et al. 2004; Warkentin et al. 2023). However, the absence of such a relationship in our results suggests that parental physiology and body condition—which dictate the provision of resources such as food—exert a more significant influence on brood sex ratios than brood size itself (Székely, Cuthill, et al. 2004; Székely, Freckleton, and Reynolds 2004; Whittingham et al. 2005; Ding et al. 2017).

### 4.4 Body Condition and Brood Sex Ratio

No significant relationship was observed between the brood sex ratio and the body mass of Black‐crowned Night Heron nestlings, which served as a proxy for body condition (for similar findings, see Ding et al. 2017). Conversely, other studies have suggested that male nestlings—which often require more energy—are expected to exhibit greater body mass near fledging when parental provisioning capacity is high (Rosivall et al. 2010; Ledwoń 2011; Banach et al. 2021; Gyarmathy et al. 2024). However, the absence of such a correlation in our results suggests that brood sex ratio biases may be driven by other parameters, such as species‐specific environmental conditions or parental quality (Ding et al. 2017; Kamiński et al. 2019).

### 4.5 Limitations and Suggestions

Due to the high costs of molecular sexing and the temporal constraints of long‐term colony monitoring, the sample size available to analyze the relationship between brood sex ratios and various ecological parameters was limited. Furthermore, the inherent challenges of capturing free‐living adults and the difficulty of accurately determining their age meant that parental morphometrics and age could not be assessed as proxies for quality or condition. Consequently, nestling morphometric traits were used as indicators of quality. However, because hatching is asynchronous, the presence of nestlings of varying sizes within a nest does not necessarily imply that smaller individuals are in poorer physical condition; this complexity precluded a more definitive analysis of the relationship between individual body condition and the brood sex ratio. Future research incorporating parental age, physiological condition, and additional environmental variables would provide valuable insights into the evolutionary mechanisms driving sex allocation in herons and other avian species, benefiting both ecologists and conservation managers.

## 5 Conclusions

The results demonstrated that nest site parameters, timing of breeding, brood size, and nestling body condition had no significant relationships with brood sex ratio of the Black‐crowned Night Heron. Since the brood sex ratio appears to be independent of these parameters, conservation and management strategies focusing on these parameters can be flexibly implemented to support nestling survival without the risk of biasing brood sex ratios. These findings suggest that the brood sex ratio in the Black‐crowned Night Heron may be primarily governed by genetic parameters or other unmeasured physiological and environmental mechanisms, highlighting the need for further research into the regulatory processes of this species.

## Author Contributions

**Seyedeh Farahnaz Vesali:** conceptualization (equal), investigation (equal), methodology (equal), project administration (equal), visualization (equal), writing – original draft (equal), writing – review and editing (equal). **Hossein Varasteh Moradi:** data curation (equal), funding acquisition (equal), methodology (equal), project administration (equal), supervision (equal), validation (equal), visualization (equal), writing – review and editing (equal). **Seyed Mehdi Amininasab:** conceptualization (equal), data curation (equal), formal analysis (equal), investigation (equal), methodology (equal), project administration (equal), supervision (equal), validation (equal), visualization (equal), writing – original draft (equal), writing – review and editing (equal). **Zarbakht Ansari Pirsaraei:** data curation (equal), investigation (equal), methodology (equal), visualization (equal).

## Funding

This work was supported by Gorgan University of Agricultural Sciences and Natural Resources.

## Ethics Statement

Nest monitoring, brood measurements, and blood sampling of Black‐crowned Night Herons were conducted in strict accordance with the approved ethical protocol (No. 8/141316, 2023, Gorgan University of Agricultural Sciences and Natural Resources) to minimize disturbance, promote animal welfare, and ensure scientific justification.

## Conflicts of Interest

The authors declare no conflicts of interest.

## Supporting information


**Table S1:** Pearson correlation coefficients among Black‐crowned Night Heron nest parameters (*n* = 18; Values indicate correlation coefficients and asterisks indicate significance levels).
**Table S2:** Pearson correlation coefficients among Black‐crowned Night Heron nestling body condition parameters (*n* = 18; Values indicate correlation coefficients and asterisks indicate significance levels).

## Data Availability

The data for this study are available on: https://doi.org/10.5061/dryad.tht76hff3.
