# Supplementary material for: The Relationship of Nest‐Site Selection Parameters, Timing of Breeding, Brood Size, and Nestling Body Condition With Brood Sex Ratio in the Black‐Crowned Night Heron Nycticorax nycticorax
Source: Ecol Evol. 2026 Jun 3;16(6):e73737. doi: 10.1002/ece3.73737 (PMC13239531; doi:10.1002/ece3.73737)
Supplement: Supplementary file 1 — Table S1: Pearson correlation coefficients among Black‐crowned Night Heron nest parameters (n = 18; Values indicate correlation coefficients and asterisks indicate significance levels). Table S2: Pearson correlation coefficients among Black‐crowned Night Heron nestling body condition parameters (n = 18; Values indicate correlation coefficients and asterisks indicate significance levels). [file ECE3-16-e73737-s001.docx]

**Supporting Information**

**TABLE S1** | Pearson correlation coefficients among Black-crowned Night Heron nest parameters (n=18; Values indicate correlation coefficients and asterisks indicate significance levels).

| **Variables** | **Variables** |  |  |  |
| --- | --- | --- | --- | --- |
|  | Nearest neighbor nest distance | Nest height above the ground | Nest depth | Circumference of the trunk at breast height |
| Nest diameter | $0.210$ | $-0.406$ | $0.835$*** | $0.030$ |
| Nearest neighbor nest distance |  | $0.330$ | $0.394$ | $0.557$* |
| Nest height above the ground |  |  | $-0.286$ | $0.389$ |
| Nest depth |  |  |  | $0.062$ |

)* P ≤ 0.05, ** P ≤ 0.01, ***P ≤ 0.001)

**TABLE S2 |** Pearson correlation coefficients among Black-crowned Night Heron nestling body condition parameters (n=18; Values indicate correlation coefficients and asterisks indicate significance levels).

| **Variables** | **Variables** |  |  |
| --- | --- | --- | --- |
|  | Body mass | Wing length | Tarsus length |
| Body length | $0.964$ *** | $0.963$ *** | $0.968$ *** |
| Body mass |  | $0.943$*** | $0.951$*** |
| Wing length |  |  | $0.973$ *** |

)* P ≤ 0.05, ** P ≤ 0.01, ***P ≤ 0.001)
